# Authors’ Reply: Empathy Cuts Both Ways in Clinical AI

**DOI:** 10.2196/110739

**Published:** 2026-10-02

**Authors:** Sang Won Bae, Tongze Zhang, Tammy Chung, Anind Dey

**Affiliations:** 1 Department of Systems Engineering, Human-Computer Interaction and Human-Centered AI Systems Lab, AI for Healthcare Lab, Charles V. Schaefer, Jr. School of Engineering and Science, Stevens Institute of Technology, 1 Castle Point Terrace, Hoboken, NJ, 07030, United States, 1 (201) 216-5687; 2 Institute for Health, Healthcare Policy and Aging Research, Rutgers Biomedical and Health Sciences, Rutgers University, Newark, NJ, United States; 3 Information School, University of Washington, Seattle, WA, United States

**Keywords:** artificial intelligence, AI, clinical decision support systems, large language models, empathy, explainable AI, trust, human factors, reliance, human-AI interaction

Sorin and Klang’s Letter to the Editor [1] regarding our evaluation [2] of an empathic clinical decision support system raises an important distinction between perceived trust and objectively warranted reliance. Emotion-adaptive communication may make accurate advice easier to understand and accept, but it could also make inaccurate advice harder to question or reject [1]. We agree that such communication should not indiscriminately increase acceptance of AI output.

Our study compared explanation delivery; it did not evaluate clinical effectiveness or appropriate reliance. The study’s empathy-enhanced and basic conditions used the same cannabis-intoxication prediction and explainable AI pipeline; the empathic condition added affect sensing and emotion-adaptive delivery. We measured perceived usability, clarity, trustworthiness, and reliability—not decision accuracy, error detection, reliance, or patient outcomes. Separately reported predictive-model performance therefore does not establish that the increase in perceived trust was warranted. Consistent with this measurement concern, Sorin et al’s systematic review [3] found that all but 1 of 12 studies assessed large language model empathy subjectively, underscoring the need for objective behavioral outcomes.

The relationships among accuracy, trust, and appropriate reliance require nuance to understand [4]. Users may consult decision support because they cannot independently determine whether advice is correct. Their ability to evaluate recommendations varies with clinical expertise, AI literacy, case difficulty, prior beliefs, and confidence—important considerations in our heterogeneous sample [2]. Objective accuracy is necessary but not sufficient for trustworthy use. Accurate information may be underused when presented confusingly, too technically, or without sufficient context [2]. Conversely, empathic delivery must not conceal uncertainty, weaken explanation fidelity, or make unsupported advice appear authoritative. Our design cannot determine whether higher trust reflected improved comprehension, persuasive delivery, or both.

Our study addressed the first communication barrier. We did not manipulate advice correctness; therefore, our data neither demonstrate nor rule out empathy-induced overreliance on AI-generated output. Participants reported that adaptation sometimes changed response focus or specificity, and we acknowledged the need for guardrails preserving clinical precision and explanation fidelity [2]. The same concern applies when an underlying prediction or recommendation is inaccurate, because inaccurate AI advice can impair clinical judgment [5].

Future studies should examine trust mechanisms beyond ratings. A useful design might, for example, vary advice correctness and communication style, hold the clinical scenario and evidentiary content constant across communication-style conditions, and assess effects across levels of clinician expertise. Outcomes could include objective comprehension, pre- and postadvice decisions, confidence calibration, verification behavior, acceptance of correct advice, rejection of incorrect advice, and both overreliance and underreliance on AI-generated output. Uncertainty communication and explanation fidelity also could be assessed.

Accordingly, our findings provide preliminary evidence that emotion-adaptive delivery can improve perceived clarity, usability, and trust; they do not establish improved decision accuracy or appropriate reliance. The goal should not be maximal perceived trust but warranted trust and appropriate reliance grounded in system performance, transparent uncertainty, faithful explanation, and users’ ability to interpret the evidence.
